# Soluble Expression of a Neo2/15-Conjugated Single Chain Fv against PD-L1 in *Escherichia coli*

**DOI:** 10.3390/cimb44010022

**Published:** 2022-01-09

**Authors:** Sun-Hee Kim, Hee-Jin Jeong

**Affiliations:** 1 Industry-Academia Cooperation Foundation, Hongik University, 2639 Sejong-ro, Jochiwon-eup, Sejong-si 30016, Korea; marian8151@hongik.ac.kr; 2 Department of Biological and Chemical Engineering, Hongik University, 2639 Sejong-ro, Jochiwon-eup, Sejong-si 30016, Korea

**Keywords:** programmed death-ligand 1, single chain Fv, Neo2/15, immunocytokine, recombinant fusion protein

## Abstract

Immunocytokines, antibody-cytokine fusion proteins, have the potential to improve the therapeutic index of cytokines by delivering the cytokine to the site of localized tumor cells using antibodies. In this study, we produced a recombinant anti-programmed death-ligand 1 (PD-L1) scFv, an antibody fragment against PD-L1 combined with a Neo2/15, which is an engineered interleukin with superior function using an *E. coli* expression system. We expressed the fusion protein in a soluble form and purified it, resulting in high yield and purity. The high PD-L1-binding efficiency of the fusion protein was confirmed via enzyme-linked immunosorbent assay, suggesting the application of this immunocytokine as a cancer-related therapeutic agent.

## 1. Introduction

The immune checkpoint blockades for the treatment of cancer have received increasing attention in novel immunotherapy. Among immune checkpoints, programmed cell death protein 1 (PD-1) and its corresponding ligand, programmed death-ligand 1 (PD-L1), are characterized and their applications as immune targets are widely established. Antibody against PD-L1 has been approved for application in clinical immunotherapy because of its effective responses in restoring the activity of exhausted T cells to recognize and destroy tumor cells [1]. Our group recently produced a single chain Fv (scFv) type antibody fragment against mouse PD-L1 (mPD-L1) in *Escherichia coli* (*E. coli*), which has high antigen-binding efficiency with a low limit of detection (LOD) [2]. As the size of scFv is approximately six times smaller than the one of full-sized antibodies, scFvs can penetrate dense tumor cells better than full-sized antibodies [3,4]. Moreover, the small size, as well as the reduced complexity of scFv, allows for rapid, low labor-intensive, and cost-effective production in *E. coli* [5,6].

Cytokines are proteins that regulate innate and adaptive immune systems and play an important role in the differentiation and polarization of immune cells into functional subtypes [7]. Notably, cytokines stimulate the activity of leukocytes which impacts the proliferative and invasive abilities of cancer cells and enhance the recruitment of the host immune system into the cancer microenvironment [8]. As cytokines are present in blood at very low concentrations with picomolar orders, recombinant cytokines have currently been generated and used as biopharmaceutical products. Neoleukin-2/15 (Neo2/15), a computationally designed mimic of IL-2, has recently been developed for effective anticancer immunotherapy [9]. IL-2 is a critical cytokine for cytotoxic and regulatory lymphocyte activation through IL-2 receptor α (IL-2Rα). However, substantial clinical toxicities have been associated with high-dose IL-2 treatment. Thus, a novel approach to circumvent this issue includes targeting IL-15, which sustains the survival of multiple cytotoxic lymphocyte subsets. IL-15 shares the heterodimer of the IL-2 receptor β- and γ-chains (IL-2Rβγc) with IL-2 and requires a distinct receptor α subcomponent (IL-15Rα). Neo2/15 was created by optimizing the functional activation of IL-2 and showed an enhanced affinity for IL-2Rβγc with no binding interface for IL-2Rα, improved heat stability, and mitigated off-target toxicities seen with IL-2 [9]. The authors demonstrated that Neo2/15 treatment expanded cytotoxic CD8+ T cells over T regulatory cells, and the combination therapy with Neo2/15 and a tumor-targeting monoclonal antibody significantly improved the tumor growth inhibition in a murine melanoma model.

To deliver cytokines to the tumor microenvironment, antibodies can be used for directing cytokines to antigens expressed on the surface of tumor cells, leading to a dramatic potentiation of the anticancer activity of cytokines. Several studies have been conducted about the combination of anti-PD-1/PDL-1 antibodies with cytokines to extend the clinical effects of PD-1/PD-L1 targeted therapies in the treatment of tumors [10,11,12]. For example, Julia EP et al. represented that the combination of Avelumab, an anti-PD-L1 antibody, with IL-2 or IL-15 enhanced the therapeutic efficacy of the antibody by increasing lytic activity against triple-negative breast cancer cells [13]. Other studies have reported a correlation between the use of tumor-specific antibody-cytokine fusions and the therapeutic efficacy, and the fusion proteins could mediate immunogenic cell death and make tumors more responsive to the action of proinflammatory biopharmaceuticals [14,15,16,17,18].

**Figure 1:**
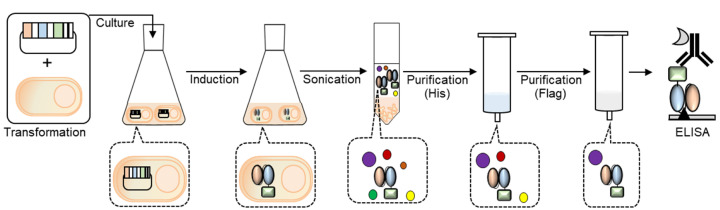
Scheme of the generation of Neo2/15-conjugated recombinant anti-PD-L1 scFv.

In this study, we combined the anti-PD-L1 scFv-encoding gene to the Neo2/15-encoding gene and expressed a recombinant Neo2/15-conjugated anti-PD-L1 scFv, which can be used for effective immunotherapy as an immunocytokine that has the potential to localize on the surface of tumor cells and to activate anticancer immunity. We established the protein purification method and confirmed the antigen-binding efficiency of the fusion protein using enzyme-linked immunosorbent assay (ELISA) (Figure 1).

## 2. Materials and Methods

### 2.1. Materials

Chemical genes were obtained from Lncbio (Seoul, Korea). Oligonucleotides and the plasmid miniprep kits were obtained from Bionics (Seoul, Korea). KOD-One DNA polymerase and the In-Fusion HD cloning kit were from Takara-Bio (Tokyo, Japan). *E. coli* SHuffle T7 Express lysY were obtained from New England Biolabs (Seoul, Korea). Talon beads were obtained from Clontech (Mountain View, CA, USA). The gravity empty column was from Bio-Rad (Daejeon, Korea). Ultrafiltration devices were obtained from Pall (Nanosep Centrifugal-3 k; Ann Arbor, MI, USA). Anti-DYKDDDDK-HRP conjugate antibody was obtained from (Biolegend, CA, USA). Recombinant mouse PD-L1 protein was obtained from Sino Biological Inc. (Beijing, China). Spin-type ultrafiltration devices were obtained from Millipore (Bedford, MA, USA). Maxi plates were obtained from SPL Life Sciences (Gyeonggi-do, Korea). HRP-conjugated anti-Flag antibody was obtained from Biolegend (San Diego, CA, USA). Other chemicals and reagents, unless otherwise indicated, were from Sigma (Seoul, Korea).

### 2.2. Gene Construction

The Neo2/15 coding gene [9] was chemically synthesized and amplified by polymerase chain reaction (PCR) using primers IL2/15 linker 2F (5′-tcccgggacctcagagtccgccacacccgaaagtcccaagaagaagatccaa-3′) and IL2/15 infusion R (5′-atgagaacccccccctgaaaagatccaactctg-3′), and KOD-plus Neo DNA polymerase. The product was ligated to pSrtCys::aPDL1scFv [2], which was amplified by PCR using PDL1 vector F (5′-ggggggggttctcatcatca-3′) and IL2/15 insertion R (5′-tacagtgcatgttcagcatgtaattggatcttcttcttgggactttcgggtgtggcgga-3′) as primers, using the In-Fusion enzyme. The PCR mixtures contained 5 μL of 10x buffer, 5 μL of 2 mM dNTPs, 3 μL of 25 mM MgSO4, 1 μL of 10 μM primer pairs, 50 ng template DNA, and 1 U enzyme, up to a volume of 50 μL with distilled water. Amplification of insert DNA was performed under the following conditions: 94 °C for 2 min; 35 cycles of 98 °C for 10 s, 54 °C for 30 s, and 68 °C for 30 s. Amplification of vector DNA was performed under the following conditions: 94 °C for 2 min; 35 cycles of 98 °C for 10 s, 49 °C for 30 s, and 68 °C for 180 s. The obtained plasmid was prepared using the plasmid miniprep system, and the entire coding-region sequence was confirmed by sequencing (Table 1).

### 2.3. Expression

SHuffle T7 Express lysY cells were transformed with each expression vector pSrtCys::aPDL1scFv-NeoIL2/15 and cultured at 37 °C for 16 h in LBA medium (LB medium containing 100 μg/mL ampicillin) and 1.5% agar. A single colony was picked and grown at 30 °C in 4 mL of LBA medium overnight, from which 1 mL was used to inoculate 100 mL of LBA medium or 2xYTA medium (2xYT medium containing 100 μg/mL ampicillin). The cells were cultured at 37 °C to an OD600 of 0.8, after which 1 mM isopropylthio-β-galactopyranoside (IPTG) was added. The solution was incubated for an additional 16 h at 37 °C for inducing, followed by centrifugation (4000× *g*, 20 min, 4 °C). The pellet was washed using 10 mL of binding buffer (50 mM phosphate buffered saline (PBS, pH 7.4)), 0.5 M sodium chloride (NaCl), 10 mM imidazole, and 5% glycerol (pH 7.4)) and resuspended in 15 mL of lysis buffer (50 mM PBS (pH 7.4), 0.5 M NaCl, 10 mM imidazole, 5% glycerol, and 1 mM PMSF), followed by sonication (50% power, 2 sec on/off).

### 2.4. Western Blot Analysis

After sonication, the sample was loaded onto the SDS-PAGE gel as a total fraction. The sonicated sample was separated through centrifugation (13,000 rpm, 30 min, 4 °C) and the supernatant was loaded onto the SDS-PAGE gel as a soluble fraction. An amount of 10 μL of protein was loaded on a 15% gel and electrophoretically transferred to PVDF membranes. Membranes were then incubated with the HRP-conjugated anti-DYKDDDK antibody.

### 2.5. Talon Purification

After the centrifugation (13,000 rpm, 30 min, 4 °C) of the sonicated sample, the supernatant was purified via the gravity purification method using 2 mL of Talon resin-packed column as follows: the supernatant was bound to beads at 4 °C, and the beads were washed with 15 mL of binding buffer followed by 60 mL of washing buffer (50 mM PBS, 0.5 M NaCl, and 20 mM imidazole). After the series addition of 1 mL of elution buffer (50 mM PBS, 0.3 M NaCl, and 250 mM imidazole), each fraction was collected using a disposable gravity column. The eluent was subjected to an ultrafiltration device (3 k), equilibrated with PBS, and concentrated to 250 μL. Protein expression and purification were confirmed by sodium dodecyl sulfate-polyacrylamide gel electrophoresis (SDS-PAGE) analysis, and protein concentration was determined on the gel using Image Lab software and using various concentrations of bovine serum albumin (BSA) as a standard (Bio-Rad) (Supplementary Figure S1). The purity of the protein was determined on the gel by dividing the area of the target protein band by the area of the total protein bands using ImageJ software (Bethesda, Maryland, USA) (Supplementary Figure S2).

### 2.6. Flag Purification

To purify the protein via Flag-tag, anti-DYKDDDDK-tagged antibody beads (100 μL) were added to the tube. After incubation at 25 °C for 1 h, the beads were washed three times with 1 mL of 1X TBS (20 mM Tris-HCl, and 0.15 M NaCl, pH 7.4). The protein was eluted from the beads by adding 500 μL of elution buffer (0.1 M Glycine-HCl, pH 3.5) three times. The eluent was immediately neutralized using 1 M Tris-HCL (pH 8.0) wash buffer at 25 °C and subjected to an ultrafiltration device (3 k or 10 k), equilibrated with PBS, and concentrated to 250 μL. Protein purification was confirmed by SDS-PAGE analysis, and the protein concentration was determined on the gel using Image Lab software and using various concentrations of BSA as a standard (Supplementary Figure S1). The purity of the protein was calculated using ImageJ software (Supplementary Figure S2).

### 2.7. Enzyme-Linked Immunosorbent Assay

The antigen-binding activity of the IL2/15-conjugated antibody fragment was confirmed by indirect ELISA. The 96-well microplate was coated with 50 μL/well of several concentrations of mPDL1 in PBS at 4 °C overnight. The plate was blocked at 25 °C for 2 h with 3% skim milk in PBST (PBS with 0.05% Tween20), washed three times with PBS, and incubated with 100 µg/mL of antibody fragments in PBST at 25 °C for 1 h. The plate was washed and incubated with a 50 μL/well of 20,000-fold diluted HRP-conjugated anti-Flag antibody in PBST at 25 °C for 1 h. The plate was then washed three times with PBS and developed with 50 μL/well TMBZ solution. After incubation for 5 min, the reaction was stopped by adding 50 μL/well of 10% sulfuric acid, and the absorbance was read using a microplate reader at 450 nm.

## 3. Results

### 3.1. Construction of Immunocytokine-Expressing Plasmid

**Figure 2:**
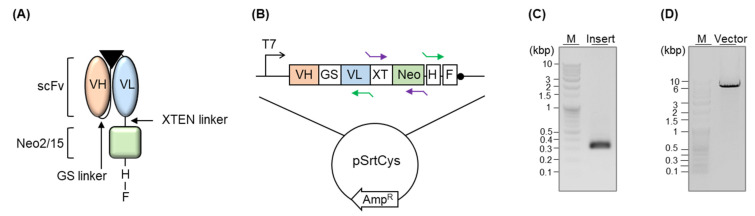
(**A**) Schematic representation of the fusion protein. The triangle indicates an antigen. (**B**) Schematic representation of the DNA plasmid. GS, XT, H, and F indicate the GS linker, XTEN linker, His-tag, and Flag-tag, respectively. Purple arrows and green arrows indicate the primer set for insert PCR and vector PCR, respectively. (**C**) Agarose gel electrophoresis analysis of DNA after the insert PCR. (**D**) Agarose gel electrophoresis analysis of DNA after vector PCR.

We conjugated the scFv-encoding gene to the Neo2/15-encoding gene via the XTEN linker and linked a His-tag followed by a Flag-tag at the 3′-terminal region of the Neo2/15 gene (Figure 2A,B). First, we performed a codon-optimization of the amino acid sequence of Neo2/15 (G2-neo2_40_1F_seq36-S11), which was designed by DeNove sequencing [9], and genetically synthesized the gene. We added the XTEN linker, a short flexible linker with no specific structure, at the 5′-terminal region of the Neo2/15 gene by PCR (Figure 2C) and linked the sequence to the 3-terminal of the scFv-expressing gene of a pSrtCys::anti-mPDL1 scFv (Figure 2D) by in-fusion ligation. We added a His-tag for purifying the protein after cytoplasmic expression, as well as for detecting it via Western blotting, followed by a Flag-tag for enabling purification as well as enzyme-linked immunosorbent assay (ELISA).

### 3.2. Expression and Purification

**Figure 3:**
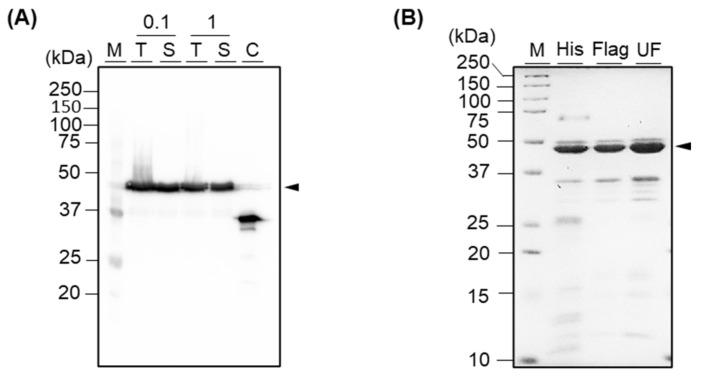
(**A**) Western blot analysis of recombinant fusion protein, which was induced with 0.1 mM or 1 mM of IPTG. M, T, S, C, and numbers indicate marker, total reagent after sonication, supernatant after centrifugation of the sonicated protein, control, and IPTG concentration, respectively. Anti-mPD-L1 scFv without Neo-IL2/15 was used as a control. (**B**) SDS-PAGE analysis of His-tag purified protein (His), His-tag- followed by Flag-tag-purified protein (Flag), and His-tag- followed by Flag-tag-purified and ultra-filtrated protein (UF). M indicates marker. Arrow indicates the target protein.

We expressed the fusion protein in *E. coli*. At that time, we induced the protein expression with 0.1 mM or 1 mM IPTG to select a more efficient IPTG concentration for obtaining a higher production yield and compared those yields via Western blot analysis. As a result, we confirmed that the protein was expressed in soluble with both concentrations of IPTG, with an expected size of 44 kDa (Figure 3A). As there was no significant difference between the two concentrations of IPTG, we decided to use a lower concentration of IPTG for inducing. Next, we purified the protein using an immobilized cobalt metal affinity chromatography gravity column, which was packed with Talon beads where His-tag-conjugated proteins were able to be attached. As a result, we obtained 99.8 μg of His-tag-purified protein with correct folding from the 100 mL shake-flask culture (Figure 3B and Figure S1). As expected, the yield was slightly lower than the yield of anti-mPD-L1 scFv, which showed a yield of 564.2 μg from a 100 mL culture in our previous study [2] because the expression yield of a large protein is usually lower than a single protein. Notably, we revealed herein the expression and purification methods for obtaining the fusion protein in soluble form, with no re-folding procedure that is labor-intensive and time-consuming.

Although a target protein was selectively captured to the beads then eluted, and most other non-target proteins were eliminated by washing, some extra bands were still observed in the SDS-PAGE gel, resulting in 62.1% of purity (Figure 3B and Figure S2). To improve the purity, we subsequently purified the protein using Flag-tag affinity chromatography. As a result, we confirmed that most extra bands were eliminated, and the purity was improved to 79.8% after the sequential purification (Supplementary Figure S2). We purified 12.4 μg of His-tag purified protein and obtained 212.7 ng of protein after Flag-tag purification (Supplementary Figure S1). To further eliminate the non-target protein with the size of around 35 kDa, we performed ultrafiltration using an MWCO 10 k column. However, as the extra band still existed, we moved forward to the next step using His-tag-purified and Flag-tag-purified proteins.

### 3.3. Confirmation of Antigen-Binding Efficiency

**Figure 4:**
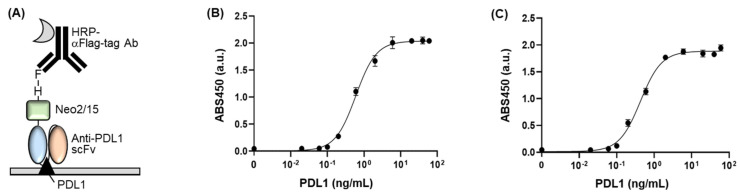
(**A**) Schematic representation of the indirect ELISA for confirming the dose-dependent PD-L1-binding efficiency of the fusion protein. (**B**) ELISA signal of Flag-tag-purified fusion protein with various concentrations of PD-L1. (**C**) ELISA signal of His-tag-purified fusion protein with various concentrations of PD-L1. Error bars represent ±1 SD (n = 3).

We examined the antigen-binding efficiency of the fusion protein by performing an indirect ELISA. We seeded commercially available mPD-L1 protein into a 96-well plate and blocked the wells using skim milk. Subsequently, we added Flag-tag-purified fusion protein as the primary antibody followed by HRP-conjugated anti-Flag antibody as the secondary antibody (Figure 4A). As a result, we confirmed antigen concentration-dependent signals of antibody, indicating that the fusion protein has a significant antigen-binding activity (Figure 4B). The EC50 value and limit of detection (LOD) value of the protein were 0.596 ± 0.032 ng/μL and 0.051 ng/μL, respectively. When we compare these values to the ones of scFv without NeoIL2/15 (EC50 of 0.325 ± 0.021 ng/μL and LOD of 0.026 ng/μL) [2], the fusion protein retained the antigen-binding activity, even linking to the large cytokine protein as the antigen-binding sites of antibody are located on the N-terminal of the antibody. This result indicates that the fusion protein binds to PD-L1 with high affinity and sensitivity, which has merit for the immunotherapeutic approach owing to the need for low amounts of the dosing-reagent and clinical sample.

Additionally, we performed ELISA using a His-tag-purified fusion protein as the primary antibody owing to confirm the effect of the non-target proteins, which have been included in the purified sample and appeared on the SDS-PAGE gel as extra bands (Figure 4C). As a result, the His-tag-purified fusion protein showed an EC50 value of 0.431 ± 0.037 ng/μL and a LOD of 0.069 ng/μL, which was similar to the Flag-tag-purified fusion protein. This result indicates that the non-target proteins might not be attached to the antigen immobilized to a plate, and thus eliminated at the washing procedure, resulting in no effect on the ELISA signal. Although an extra protein with the size of around 35 kDa still existed in the sample after Flag-tag purification, we should mention that it was not critically affected by the antigen-binding affinity of the fusion protein because the EC50 and LOD values of the fusion protein were similar to the ones of the non-Neo2/15 conjugated scFv.

## 4. Discussion

In this study, we generated Neo2/15-conjugated anti-PD-L1 scFv using an *E. coli* expression system and demonstrated its antigen-binding efficiency with nanogram orders of EC50 and LOD values. The expression and purification conditions for obtaining the fusion protein in a soluble form with high yield and purify can be useful for its cost-effective large-scale production using *E. coli*. Although additional purification methods, such as size exclusion chromatography and ion-exchange chromatography can be used to further improve the purity, those methods cause a high loss of protein sample and need high-cost equipment. Although it was not able to directly confirm whether the extra protein in the sample affected the antigen-binding efficiency, it was certain that the EC50 and LOD values of the fusion protein were similar to the ones of anti-PD-L1 scFv, which showed higher purity than the Neo2/15-conjugated anti-PD-L1 scFv [2], indicating the extra protein might not affect the binding ability of the fusion protein to antigen.

As we genetically linked a Neo2/15 to an antibody against PD-L1 on the tumor cells, the specificity and selectivity of Neo2/15 localization to tumor cells can be increased. Moreover, we used a small-sized antibody fragment instead of a full-sized antibody in intact IgG format. Therefore, the fusion protein consists of two simple functions, the therapeutic activity of the cytokine and antigen-binding efficacy of the antibody. Indeed, in contrast to IgG-based immunocytokine (approximately 180 kDa), the scFv-based immunocytokine (approximately 50 kDa) can relatively easily penetrate dense tumor cells and rapidly be cleared from circulation. Based on these points, we expect that this fusion protein can be used as an attractive cancer-related therapeutic immunocytokine.

## Figures and Tables

**Table 1:** Nucleotide and amino acid sequences of Neo2/15-conjugated anti-PDL1 scFv with tags. Underline: OmpA signal peptide, normal text: anti-PDL1 scFv, underlined bold: XTEN linker, italic: Neo2/15, bold italic: GS linker, underlined italic: His-tag, underlined bold italic: Flag-tag.

	Sequence
Nucleotide (5′-3′)	atgaaaaagacagctatcgcgattgcagtggcactggctggtttcgctaccgtggcccaggcggccctg actcagccgtcctcggtgtcagcaaacctgggaggaaccgtcaagatcacctgctccgggggtagtggc agctacggctggtatcagcagaaggcacctggcagtgcccctgtcagtctgatctatgacaacaccaac agaccctcggacatcccttcacgattctccggtgccctatccggctccacagccacattaaccatcactgg aggtccaagccgaggacgaggctgtctattactgtgggagcagggacagcagtaatgctggttctgtatt tggggccgggacaaccctgaccgtcctaggtcagtcctctagatcttccggcggtggtggcagctccgg tggtggcggttccgccctgacgttggacgagtccgggggcggcctccagacgcccggaggagcgct cagcctcgtctgcaaggcctccgggttcaccttcagtgaccgtggcatgcactgggtgcgacaggcgcc cggcaaggggctggagtgggtcggtgctattagcaggagagggagtaccacaacttacgcacccgcgg tgaagggccgtgccaccatcacgagggacaacgggcagagcacagtgaggctgcagctgaacaacct cactgctgaggacaccgccacctacttctgcgccaaaaatgatgattctgtcggtatagtgactacttctac tatcgacgcatggggccacgggaccgaagtcatcgtctcctccactagtggccaggccggccag**agcg** **gcagcgagactcccgggacctcagagtccgccacacccgaaagt***cccaagaagaagatccaattacatgct* *gaacatgcactgtatgacgccttgatgatcttgaatattgtcaaaaccaactcgccgccggcagaagagaagcttg* *aagattatgcatttaattttgaacttatccttgaggaaattgcacgtttattcgaaagtggtgatcaaaaagatgaag* *ccgagaaggccaagcgcatgaaagagtggatgaaacgtatcaagaccaccgcttcagaggatgagcaggagga* *gatggcgaacgcgattattacaatcctgcagagttggatcttttca**ggggggggttct**catcatcatcatcatcat**g*** ***gcggatccgactacaaggacgacgatgacaaa***
Amino acid (N′-C′)	MKKTAIAIAVALAGFATVAQAALTQPSSVSANLGGTVKITCSGGSGSY GWYQQKAPGSAPVSLIYDNTNRPSDIPSRFSGALSGSTATLTITGVQAE DEAVYYCGSRDSSNAGSVFGAGTTLTVLGQSSRSSGGGGSSGGGGSAL TLDESGGGLQTPGGALSLVCKASGFTFSDRGMHWVRQAPGKGLEW VGAISRRGSTTTYAPAVKGRATITRDNGQSTVRLQLNNLTAEDTATYFC AKNDDSVGIVTTSTIDAWGHGTEVIVSSTSGQAGQ**SGSETPGTSESAT** **PES**PKKKIQLHAEHALYDALMILNIVKTNSPPAEEKLEDYAFNFELILE EIARLFESGDQKDEAEKAKRMKEWMKRIKTTASEDEQEEMANAIITIL QSWIFS***GGGS****HHHHHH**GGSDYKDDDDK***

## Supplementary Materials

The following are available online at https://www.mdpi.com/article/10.3390/cimb44010022/s1. Figure S1: (A) Confirmation of the folding of His-tag purified protein by comparing the mobility shift of denatured (reduced using DTT and heating) and native (non-reduced) proteins on the gel. (B–D) Measurement of the concentration of proteins using Image Lab software. A standard curve was generated using a series diluted bovine serum albumin (BSA) as a standard protein. Next, each volume of the target band (BSA or fusion protein) was estimated using the Image Lab software and the absolute quantity of target protein was calculated using a standard curve. Figure S2: Measurement of the purity of proteins using ImageJ software. Each volume of the bands of protein was estimated using the ImageJ software (A,B) and the purity was calculated by (area of target band)/(total area of whole bands appeared on the gel) × 100 (C).
