# Supplementary material for: Soluble Expression of a Neo2/15-Conjugated Single Chain Fv against PD-L1 in Escherichia coli
Source: Curr Issues Mol Biol. 2022 Jan 9;44(1):301–8. doi: 10.3390/cimb44010022 (PMC8929057; doi:10.3390/cimb44010022)
Supplement: Supplementary file 1 [file cimb-44-00022-s001.zip › cimb-1508316-supplementary.pdf]

Supplementary Materials:

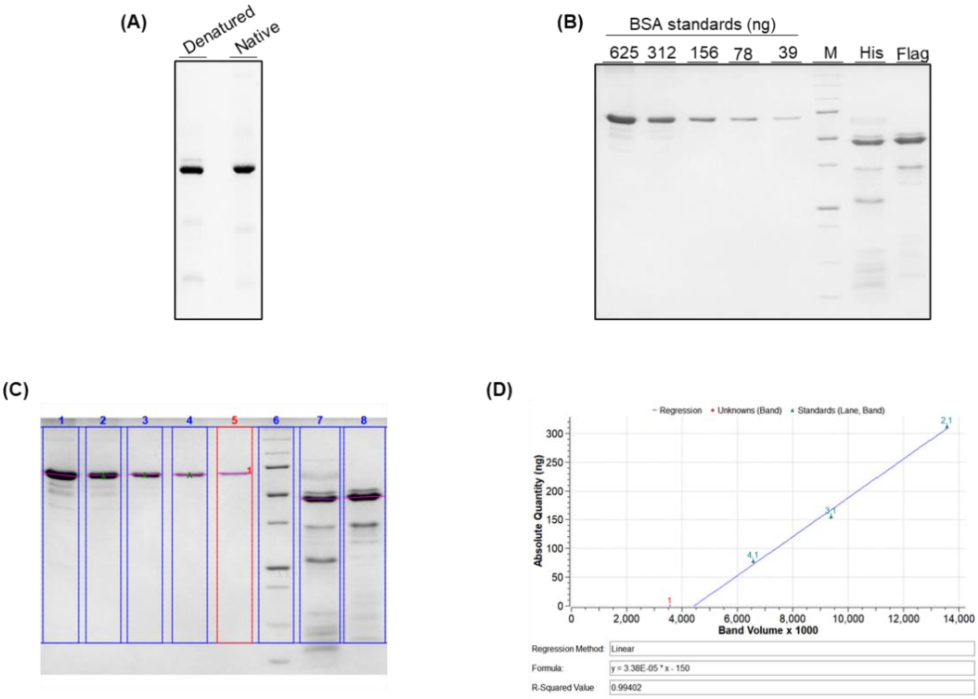

Figure S1: (A) Confirmation of the folding of His-tag purified protein by comparing the mobility shift of denatured (reduced using DTT and heating) and native (non-reduced) proteins on the gel. (B–D) Measurement of the concentration of proteins using Image Lab software. A standard curve was generated using a series diluted bovine serum albumin (BSA) as a standard protein. Next, each volume of the target band (BSA or fusion protein) was estimated using the Image Lab software and the absolute quantity of target protein was calculated using a standard curve.

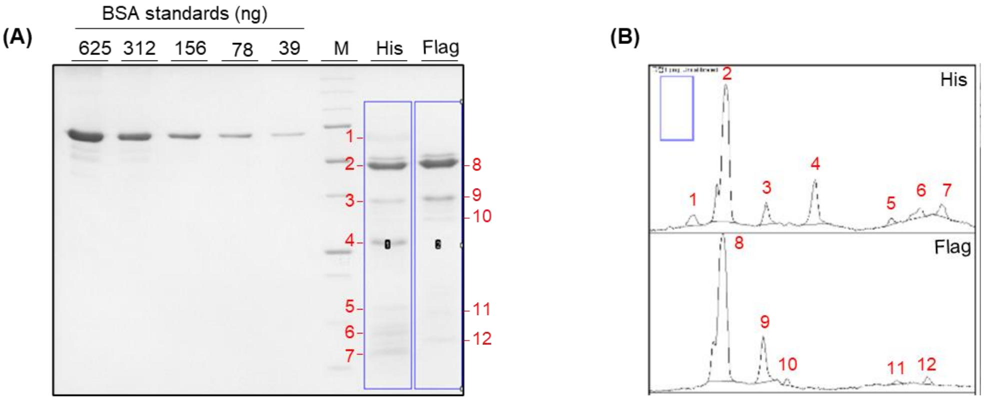

(C) His-tag purification

Band	1	**2**	3	4	5	6	7	Total
Area	366.8	**5099.9**	521.3	1339.7	144.6	375.6	363.3	8214.2
%	4.5	**62.1**	6.3	16.3	1.8	4.6	4.4	100

Flag-tag purification

Band	**8**	9	10	11	12	Total
Area	**6389.4**	1219.8	126.7	86.6	183.0	8005.5
%	**79.8**	15.2	1.6	1.1	2.3	100

Figure S2: Measurement of the purity of proteins using ImageJ software. Each volume of the bands of protein was estimated using the ImageJ software (A, B) and the purity was calculated by (area of target band)/(total area of whole bands appeared on the gel) × 100 (C).
